# Structure and Antibacterial Activity of Ambobactin, a New Telomycin-Like Cyclic Depsipeptide Antibiotic Produced by *Streptomyces ambofaciens* F3

**DOI:** 10.3390/molecules200916278

**Published:** 2015-09-09

**Authors:** Shaopeng Wei, Wenhao Zhang, Zhiqin Ji

**Affiliations:** College of Plant Protection, Northwest A & F University, Yangling 712100, Shanxi, China; E-Mails: weishaopeng@nwsuaf.edu.cn (S.W.); zhangwenhao@nwsuaf.edu.cn (W.Z.)

**Keywords:** cyclic depsipeptide, telomycin-like, *Streptomyces ambofaciens* F3, antibacterial activity

## Abstract

A new telomycin-like cyclic depsipeptide, ambobactin (**1**), was isolated from the metabolites of *Streptomyces ambofaciens* F3, an endophyte of *Platycladus orientalis*. Its structure was elucidated on the basis of extensive spectroscopic analysis and advanced Marfey’s method. Ambobactin is structurally related with telomycin, except that the configuration of the 3-methyltryptophanes in their structures is different. It exhibited strong antibacterial activity against both Gram-positive and Gram-negative bacteria. Furthermore, this investigation revealed that *S. ambofaciens* F3 is a new producer of telomycin-like antibiotics.

## 1. Introduction

Cyclic peptides produced by microorganisms are polypeptide chains that adopt cyclic ring structures. Over the decades, a large number of natural cyclic peptides have been isolated and identified by chemists, and some of them exhibit a variety of bioactivities, including immuno-suppressive, antibacterial, antifungal and antitumor activities [1,2,3]. Several naturally occurring cyclic peptides, such as cyclosporin A and vancomycin, have been used in the clinic [4]. Telomycin, an antibacterial cyclic depsipeptide, was firstly isolated by Misiek *et al.* from the fermentation broth of an unidentified *Streptomyces* [5]. This natural undecapeptide consists of aspartic acid, serine, threonine, *allo*-threonine, alanine, glycine, *cis*- and *trans*-3-hydroxyproline, β-hydroxyleucine, β-methyltryptophan and α,β-didehydrotryptophan [6]. In recent years, telomycin has also been isolated from the metabolites of *Streptomyces canus* and *Micromonospora schwarzwaldensis* [7,8]. Besides, antibiotic LL-AO341β1, an analogue of telomycin, was also obtained as a product of *Steptomyces canidus* in 1993 [9,10]. Telomycin and antibiotic LL-AO341β1 share certain structural features. For example, both of them contain the rare naturally occurring β-methyltryptophan, hydroxylated proline and leucine, and all their constituent amino acids display l-configurations [11]. This type of cyclic depsipeptides exhibited selective antibacterial activity against Gram-positive bacteria and their primary target is the cytoplasmic membrane [12].

In a program of screening antimicrobial agents from microorganism, we isolated a cyclic depsipeptide (ambobactin, **1**) from the culture broth of *Streptomyces ambofaciens* F3, an endophyte of *Platycladus orientalis*. The results of NMR, HR-TOF-MS and ESI-MS/MS analysis revealed that the composition and sequence of ambobactin was identical with those of telomycin. The absolute configuration of the constituent amino acids in ambobactin was further determined using the advanced Marfey’s method. It appears that the 3-methyltryptophan in ambobactin has a d-configuration, whereas all the constituent amino acids of telomycin have l-configurations. This implied that ambobactin is a novel cyclic depsipeptide different from telomycin. Here we report the isolation, structural elucidation and antibacterial activities of this novel telomycin-like cyclic depsipeptide.

## 2. Results and Discussion

### 2.1. Structural Elucidation

Ambobactin (**1**) was obtained as a white powder. The IR spectrum of **1** exhibited the typical absorption bands of amides at 1643 cm^−1^. Its molecular formula was established as C_59_H_77_N_13_O_19_ by HRMS (*m*/*z* 1272.5549, [M + H]^+^; calcd for C_59_H_78_N_13_O_19_, 1272.5537). The ^13^C-NMR and DEPT spectra (Table 1) of **1** showed the presence of 59 carbon signals, which were recognized as six methyls, seven methylenes, sixteen sp^3^ methines, eleven sp^2^ methines, seven sp^2^ quaternary C-atoms, and twelve carbonyl carbons. ^1^H and ^13^C chemical shift assignments were made by standard 1D and 2D NMR techniques, such as DEPT, HSQC, ^1^H-^1^H COSY and HMBC. The long-range couplings (HMBC) from the methyl protons (δ 1.05) to methine carbon (δ 48.5) and carbonyl carbon (δ 172.4), as well as the cross peak between methine proton (δ 4.45) and NH (δ 7.73), revealed the presence of alanine. The correlation between methyl protons (δ 0.79, 0.84) and methine carbons (δ 29.2, 75.2) observed in HMBC spectrum and the cross peak between two methine protons (δ 3.32, 4.49) indicated the presence of 3-hydroxyleucine (3-HyLeu). The signals observed at δ 4.46, 4.99 and 1.15 in ^1^H-NMR spectrum were assigned to the α, β, and λ protons of one threonine (Thr_1_) based on the HSQC and HMBC spectra. Another threonine (Thr_2_) could also be recognized according to the correlation signals of 2D NMR. The characteristic signals of ten aromatic methines and six aromatic quaternary carbons revealed that ambobactin incorporated two 3-substituted-indole moieties. The correlations of the methyl protons (δ 1.24) with C_2_ (δ 60.2), C_3_ (δ 33.2) and C_4_ (δ 117.5) in HMBC spectrum, as well as the cross peak between the methyl protons (δ 1.24) and H_3_ (δ 3.65), indicated the presence of 3-methyltryptophan. Meanwhile, the α,β-dehydrotryptophan moiety was established by the correlations of sp^2^ methine proton (δ 7.46) with C_10a_ (δ 127.7) and carbonyl carbon (δ 163.9). Similarly, aspartic acid, serine, glycine and 3-hydroxyproline were detected by their expected spin systems. Because the chemical shifts of several α-H are too similar to be distinguished, or no correlations were observed between α-H and carbonyl carbons in HMBC spectrum, some carbonyl carbons were impossible to assign. The NMR data of ambobactin are similar to those reported in the literature for telomycin except for the variation of chemical shifts of several carbon and hydrogen atoms [7].

**Table 1:** NMR data for ambobactin (**1**) recorded in DMSO-*d*_6_.

	Ambobactin (1)				Telomycin	
	δ_C_ (ppm) *	δ_H_ (ppm, *J* = Hz) *	HMBC	^1^H, ^1^H-COSY	δ_C_ (ppm) *	δ_H_ (ppm) *
Aspartic Acid (Asp)						
COOH	170.6				170.07	
C_α_	51.5	3.93 (m)	170.6	2.33, 2.74	48.65	4.17
C_β_	38.3	2.33 (d, *J* = 10.5 Hz)	168.8	3.93	34.24	2.82
		2.74 (t, *J* = 10.5 Hz)	168.8			
CO_γ_	168.8				168.55	
Serine (Ser)						
CO	169.0				170.70	
C_α_	57.8	4.11 (m)	169.0	3.64, 3.74, 8.67	54.00	4.61
C_β_	61.5	3.64, 3.74 (m)		4.11, 4.46	61.66	3.46, 3.68
NH		8.67 (s)		4.11		8.43
OH		4.46 (s)		3.64, 3.74		
Threonine_1_ (Thr_1_)						
CO	168.9				168.28	
C_α_	57.9	4.46 (m)	168.9	4.99, 7.76	58.3	4.25
C_β_	71.2	4.99 (m)	-	4.46, 1.15	71.0	4.98
C_γ_	15.9	1.15 (d, *J* = 6.0 Hz)	57.9, 71.2	4.99	15.77	1.23
NH		7.76 (d, *J* = 7.0 Hz)		4.46		8.10
3-Hydroxy-l-Proline (3-HyP_1_)						
CO	171.3					
C_2_	67.5	4.19 (m)	73.6, 171.3	4.21	66.8	4.21
C_3_	73.6	4.21 (m)		1.96, 2.16, 4.19, 5.78	73.07	4.22
C_4_	33.9	1.96, 2.16 (m)		3.52, 3.82, 4.21	33.32	2.00, 2.19
C_5_	44.9	3.52, 3.82 (m)		1.96, 2.16	44.32	3.49, 3.83
OH		5.78 (s)		4.21		
α,β-Dehydrotryptophan (ΔTry)						
CO	163.9				163.57	
C_2_	122.9	-			122.17	
C_3_	122.9	7.46 (s)	127.7, 163.9	7.62, 7.08	122.67	
C_4_	109.0	-			108.38	
C_5_	128.2	7.95 (s)	109.0, 127.7, 135.9	11.72	127.67	7.95
C_6a_	135.9	-			135.34	
C_7_	112.3	7.38 (d, *J* = 8.2 Hz)	120.6, 127.7	7.14	111.73	7.38
C_8_	122.5	7.14 (t, *J* = 7.6 Hz)	118.1, 127.7, 135.9	7.38	121.97	7.13
C_9_	120.6	7.08 (t, *J* = 7.6 Hz)	112.3	7.62	120.08	7.08
C_10_	118.1	7.62 (d, *J* = 8.0 Hz)	109.0, 122.5, 127.7, 135.9	7.08	117.54	7.60
C_10a_	127.7	-			127.67	
6-NH		11.72 (s)	109.0, 127.7, 135.9			11.70
NH		10.08 (s)	163.9, 122.5, 128.2			10.14
3-Methyl-Tryptophan (3-MeTry)						
CO	171.8					
C_2_	60.2	4.46 (m)	33.2, 171.8	7.56, 8.69	60.0	4.47
C_3_	33.2	3.65 (m)	112.0	1.24, 4.46	32.49	3.61
C_4_	117.5	-	-		116.6	
C_5_	123.3	7.08 (s)	112.0, 117.5, 126.3, 137.2	10.75	122.87	
C_6a_	137.2	-	-		136.56	
C_7_	112.0	7.28 (d, *J* = 8.2 Hz)	126.3, 118.7	10.75, 7.00	111.43	7.27
C_8_	121.2	7.00 (t, *J* = 7.6 Hz)	119.5, 121.2, 126.3, 137.2	7.28	120.63	7.01
C_9_	118.7	6.93 (t, *J* = 7.6 Hz)	112.0, 126.3	7.56	118.17	6.92
C_10_	119.5	7.56 (d, *J* = 8.2 Hz)	117.5, 121.2, 126.3, 137.2	6.93	119.01	7.58
C_10a_	126.3	-	-		125.56	
C_11_	19.8	1.24 (d, *J* = 7.0 Hz)	33.2, 60.2, 117.5	3.65	18.37	
6-NH		10.75 (s)	117.5, 126.3, 137.2	7.08, 7.28		10.75
NH		8.69 (s)				7.74
3-Hydroxy-Leucine (3-HyLeu)						
CO	171.6					
C_α_	52.4	4.49 (m)	171.6	3.32, 6.62	51.60	4.54
C_β_	75.2	3.32 (m)	-	1.94, 4.49, 4.74	75.18	3.29
C_γ_	29.2	1.94 (m)	-	0.79, 0.84, 3.32	28.78	1.94
C_δ1_	20.6	0.84 (d, *J* = 6.6 Hz)	16.5, 29.2, 75.2	1.94	19.63	0.87
C_δ2_	16.5	0.79 (d, *J* = 6.6 Hz)	20.6, 29.2, 75.2	1.94	17.19	0.84
OH		4.74 (s)		3.32		
NH		6.62 (s)		4.49		
3-Hydroxy-l-Proline (3-HyP_2_)						
CO	169.4					
C_2_	62.2	4.67 (m)	169.4	4.71	63.02	4.61
C_3_	69.5	4.71 (m)		1.76, 2.03, 4.67, 4.49	69.35	4.61
C_4_	32.1	1.76, 2.03 (m)		3.74, 3.93, 4.71	33.43	1.90
C_5_	45.0	3.74, 3.93 (m)		1.76, 2.03	45.25	3.61
OH		4.49 (s)		4.71		
Glycine (Gly)						
CO	169.1					
C_α_	41.5	3.80, 4.44 (m)	169.1	8.86	41.0	3.84, 4.42
NH		8.86 (s)		3.80, 4.44		8.66
Alanine (Ala)						
CO	172.4				171.4	
C_α_	48.5	4.45 (m)	172.4	1.05, 7.73	47.85	4.45
C_β_	17.5	1.05 (d, *J* = 6.0 Hz)	48.5, 172.4	4.45	17.05	1.02
NH		7.73 (d, *J* = 7.0 Hz)		4.45		8.05
Threonine_2_ (Thr_2_)						
CO	169.3					
C_α_	58.8	4.09 (m)	67.2, 169.3	3.82, 7.42	57.22	4.22
C_β_	67.2	3.82 (m)		1.07, 4.09	66.91	3.64
C_γ_	21.5	1.07 (d, *J* = 6.0 Hz)	67.2, 58.8	3.82	20.65	1.00
NH		7.42 (s)		4.09		7.53

* ^1^H and ^13^C measured at 500 and 125 MHz, respectively. NMR data of telomycin was also measured in DMSO-*d*_6_ [7].

The biggest chemical shift differences were observed in the aspartic acid and serine residues, where the chemical shifts of C_α_ and C_β_ of the aspartic acid residue in ambobactin were δ 51.5 and δ 38.3, whereas the corresponding values in telomycin were δ 48.65 and δ 34.24, and the chemical shifts of C_α_ of the serine residues in ambobactin and telomycin were δ 57.8 and δ 54.00, respectively.

**Figure 1:**
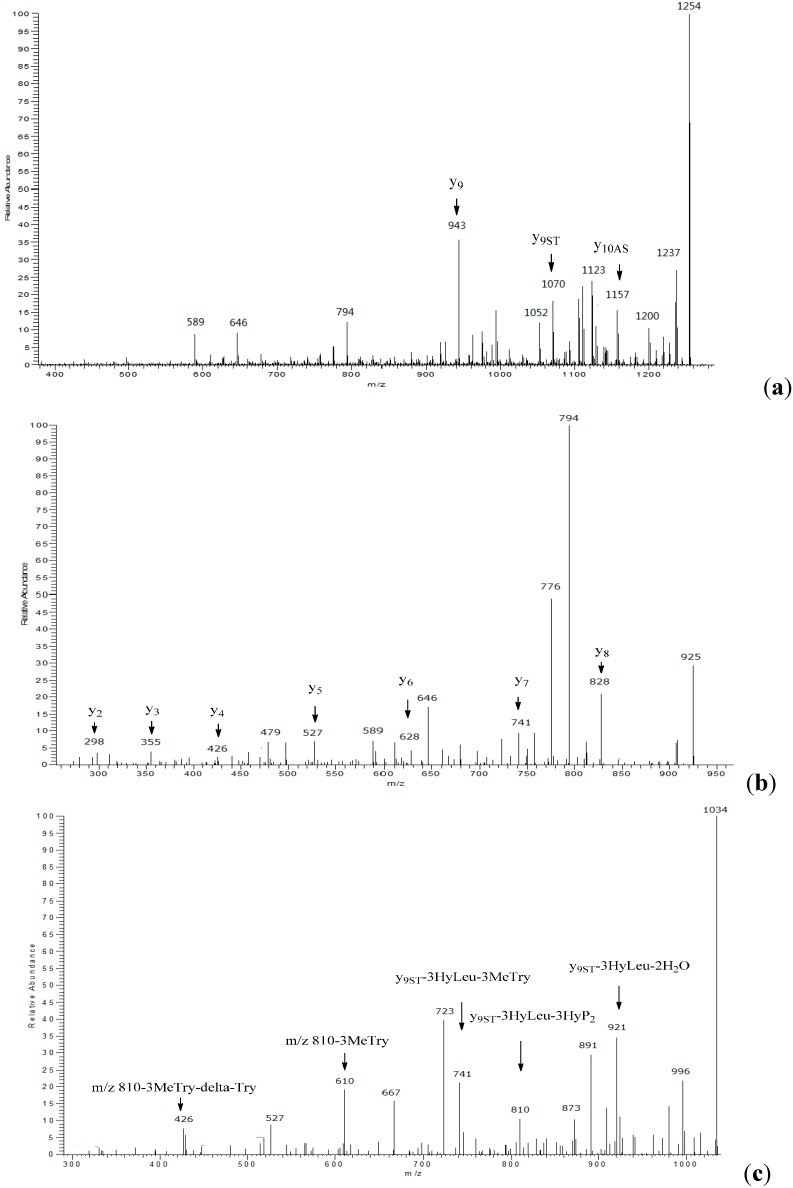
Tandem MS spectra of characteristic ions. (**a**) Product ions of *m*/*z* 1272; (**b**) Product ions of *m*/*z* 943; (**c**) Product ions of *m*/*z* 1070.

**Figure 2:**
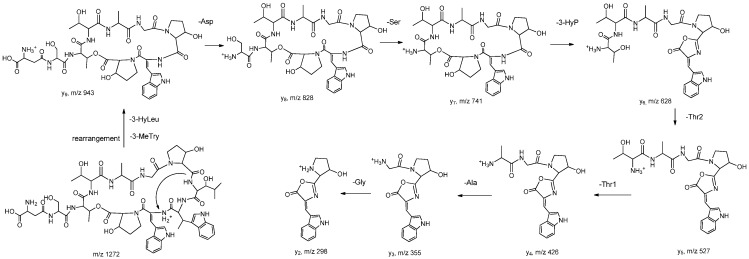
Fragmentation pathway of the *m*/*z* 943 ion.

**Figure 3:**
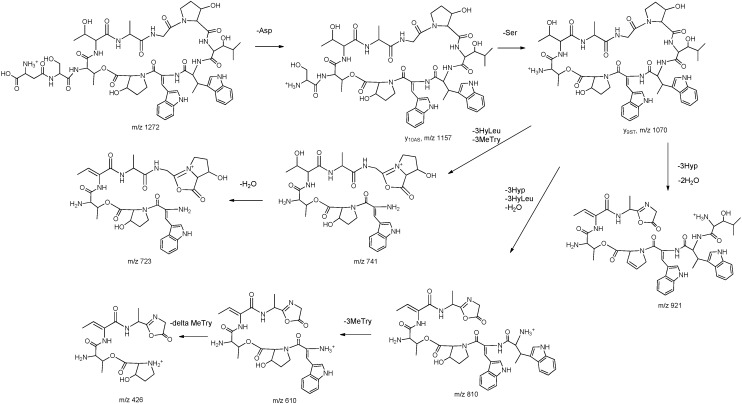
Fragmentation pathway of the *m*/*z* 1070 ion.

**Figure 4:**
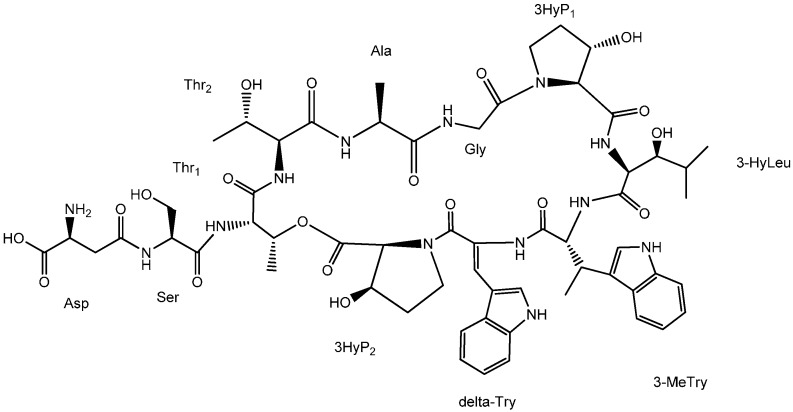
Structure of ambobactin.

Tandem mass spectroscopy has become an important and frequently used technique for determining the amino acid sequence of peptides [13]. Product ions observed in MS/MS spectrum of peptides are usually formed by the cleavage of peptide bonds and loss of small neutral species like water, ammonia, *etc.*, but only b and y ions, produced by the cleavage of peptide bonds, provide structural information about the amino acid sequence [14]. The sequence linkage of the amino acids in ambobactin could be readily determined by comparison of the characteristic ions in the spectra of **1** with the structure of telomycin. The MS/MS spectrum of **1** was illustrated in Figure 1. As shown in Figure 1a, the fragments observed at *m*/*z* 1157 and 1070 are y_10AS_ and y_9ST_ ions, and they are produced by the loss of aspartic acid and serine residues in sequence. It’s regretable that no subsequent y ions were observed in the MS/MS spectrum. The predominant *m*/*z* 943 fragment was a rearrangement y_9_ ion produced by the simultaneous loss of 3-methyltryptophan (−200 Da) and α,β-dehydrotryptophan (−184 Da) from the quasi-molecular ion. To obtain more structural information about the amino acid sequence, the *m*/*z* 943 ion was selected as parent ion to perform tandem MS analysis, and its product ions are illustrated in Figure 1b. As shown in Figure 1b and Figure 2, a series of y ions such as y_8_ (*m*/*z* 828), y_7_ (*m*/*z* 741), y_6_ (*m*/*z* 628), y_5_ (*m*/*z* 527), y_4_ (*m*/*z* 426), y_3_ (*m*/*z* 355), y_2_ (*m*/*z* 298) were observed in the spectrum. Meanwhile, the y_9ST_ ion (*m*/*z* 1070) was also selected as the precursor ion for tandem MS analysis. As shown in Figure 1c and Figure 3, the predominant ions were produced by the loss of amino acid and water simultaneously, whereas the expected sequential b or y ions were not observed in the spectrum, so the sequence linkage of the amino acids was finally established by analyzing the fragments individually. The ion observed at *m*/*z* 921 was produced by the loss of 3-hydroxyproline (−113 Da) and two molecules of water (−36 Da). The *m*/*z* 810 ion was formed by the loss of 3-hydroxyleucine (−129 Da), 3-hydroxyproline (−113 Da) and water (−18 Da), and it produced ions *m*/*z* 610 and 426 by the loss of 3-methyltryptophan (−200 Da) and α,β-dehydrotryptophan (−184 Da) in sequence. The fragment observed at *m*/*z* 741 was a b_7_ ion produced by the simultaneous.loss of 3-hydroxyleucine and 3-methytryptophan from the parent ion. These results indicated that the amino acid sequence of **1** was identical to that of telomycin (Figure 4). Furthermore, the amino acid sequence could also be justified by the correlation signal between the amide protons and α-H of adjacent amino acid residues in NOESY spectrum. For example, the presence of a segment, Thr_2_-Thr_1_-Ala-Gly, was deduced from NOESY cross-peaks for αH-Thr_2_/NH-Thr_1_, αH-Thr_1_/NH-Ala, and αH-Ala/NH-Gly.

The absolute configurations of the amino acids were determined by using the advanced Marfey method [15]. The hydrolysate of **1** was analyzed by LC/DAD/MS after derivatization with l-FDAA and d-FDAA, respectively. According to the principle of the advanced Marfey method, the relationship between the l-FDAA derivative of d-amino acid (d-l type) and that of l-amino acid (l-l type) is diastereomeric, and this pair of diastereoisomers has different retention times on chromatogram. On the other hand, the l-d and d-l type derivatives, as well as the l-l and d-d type derivatives, are enantiomers, and each pair of enantiomers has the same retention time. The selected ion chromatogram (SIC) of d- and l-FDAA derivatives of the constituent amino acids of **1** was depicted as Figure S1, Figure S2 and Figure S3, and their retention times were listed in Table 2. As shown in Table 2, the l-FDAA derivatives of aspartic acid, serine, 3-hydroxyleucine, threonine, alanine and 3-hydroxyproline were eluted before their corresponding d-FDAA derivatives, whereas only the pair of 3-methyltryptophan derivatives exhibited a different elution order. According to the validated separation mechanism, the retention times of l-d and d-l type derivatives are shorter than those of l-l and d-d type derivatives. Thus, it could be deduced that 3-methyltryptophan has a d-configuration, and all the other abovementioned amino acids have l-configurations. However, the stereochemistry of C_β_ in several amino acid residues could not be ascertained by the advanced Marfey method. The observed cross signals between H_α_ and H_β_ of 3-hyp_2_ in NOESY spectrum revealed that it was *cis*-3-hydroxyproline, and 3-hyp_1_ was determined as *trans*-3-hydroproline because no cross peak was observed. Similarly, Thr_2_ was determined as *allo*-threonine based on NOESY spectrum. All of them are identical with those of telomycin. The stereochemistry of C_β_ in Thr_1_, 3-hyLeu and 3-methyl-tryptophan was not determined based on the above methods, so we suppose they might have the same configuration with those of telomycin by comparison of the reported natural analogues.

**Table 2:** Retention times of l- and d-FDAA derivatives of the constituent amino acids.

Derivatives	Time (min)						
	Asp	Ser	3-HyLeu	Thr	Ala	3-HyP	3-MeTry
L-FDAA	10.62	7.12	21.36	7.93	18.86	10.47	44.11
D-FDAA	12.21	7.55	34.95	8.68	26.99	12.13	41.59

### 2.2. Antibacterial Activity

As shown in Table 3, ambobactin exhibited strong antibacterial activity against *B. subtilis*, *E. coli*, *E. carotovora*, *P. syringae*, *A. solanacearum* and *X. oryzae*, and their MIC values are no more than 6.25 μg/mL. Ambobactin also exhibited equivalent antibacterial activity against *B. cereus* and *S. aureus*. In general, the bioassay results indicated that most of the tested Gram-negative bacteria were sensitive to ambobactin except for *P. aeruginosa*.

**Table 3:** Antibacterial activity of ambobactin (**1**).

Compounds	MIC (μg/mL)								
	BS	BC	SA	EC	PA	ErC	PS	AS	XO
Ambobactin	3.13	25	25	6.25	＞100	6.25	6.25	6.25	6.25
Ampicillin	1.56	6.25	6.25	3.13	＞100	6.25	6.25	6.25	6.25

BS, *Bacillus subtilis*; BC, *Bacillus cereus*; SA, *Staphyloccocus aureus*; EC, *Escherichia coli*; PA, *Pseudomonas aeruginosa*; ErC, *Erwinia carotovora*; PS, *Pseudomonas syringae* pv. *actinidiae*; AS, *Alstonia solanacearum*; XO, *Xanthomonas oryzae* pv. o*ryzae*.

## 3. Experimental Section

### 3.1. General Experimental Procedures

Melting points were measured on a WPR apparatus and are uncorrected (Shanghai Jingke Co., Shanghai, China). IR spectra were recorded on a Nicolet FT-IR 750 spectrometer (Thermo Fisher Scientific, Waltham, MA, USA). UV spectra were obtained using a Shimadzu UV-2401A spectrometer (Shimadzu Co., Kyoto, Japan). Optical rotations were measured with a Horiba SEPA300 polarimeter (Horiba Ltd., Kyoto, Japan). The high resolution mass spectrum (HRMS) was obtained using an LC/MSD TOF instrument (Agilent Technologies Co., Santa Clara, CA, USA) equipped with an APCI source. Direct tandem mass spectrometric analysis of peptide was performed on a Finnigan LCQ Advantage ion-trap mass spectrometer (Thermo Fisher Co.) equipped with an ESI source. The LC-UV/MS/MS analysis of the derivatives of degraded amino acids was performed on the Finnigan LCQ Advantage ion-trap mass spectrometer (Thermo Fisher Co.) coupled with Surveyor LC pump and diode array detector. ^1^H-, ^13^C-NMR, DEPT, HSQC, HMBC, ^1^H,^1^H-COSY and NOESY spectra were obtained on Bruker Avance III-500 NMR spectrometer (Bruker Daltonics Inc., Rheinstetten, Germany). Chemical shifts were referenced to residual solvent signals. 1-Fluoro-2,4-dinitrophenyl-5-L-alanineamide (l-FDAA) was purchased from TCI (TCI Co., Shanghai, China). d-FDAA was synthesized by the reaction of 1,5-difluoro-2,4-dinitrobenzene (DFDNB) and d-Ala-NH_2_ according to a literature method [16]. Other reagents were commercially obtained.

### 3.2. Microorganism and Fermentation

The production strain *Streptomyces ambofaciens* F3 was isolated from the roots of a *Platycladus orientalis* sample collected on the campus of Northwest A&F University, Shaanxi Province, China, and identified by its morphology and 16S rRNA gene sequence (accession number: KT368940). The voucher specimen of this streptomycete was deposited at the Institute of Pesticide Science, Northwest A & F University, China.

The spores of *S. ambofaciens* F3 grown on Gause’s No. 1 agar were used to inoculate into a 250 mL flask containing 50 mL of a sterile seed medium consisting of glucose 0.8%, soluble starch 0.8%, beef extract 0.6%, peptone 1.0%, and NaCl 0.5%, pH 7.2. The flask was shaken on a shaker at 180 r.p.m. for 24 h at 28 °C. Ten milliliters of the seed culture were transferred to 250 mL flasks containing 50 mL of a sterile producing medium consisting of glucose 3.0%, millet steep liquor 1.0%, peptone 1.5%, NaCl 0.5%, and CaCO_3_ 0.5%, pH 7.2. Fermentation was carried out at 180 r.p.m. for 7 days at 28 °C on a rotary shaker.

### 3.3. Extraction and Isolation

The culture of 25 liters was filtered with cheesecloth to separate the medium and culture liquid. The filtrate was absorbed onto HPD100 macroporous resin (1.0 kg, Baoen Co. Ltd, Hebei, China), and then eluted with methanol. After the methanol was evaporated under vacuum, and the concentrate (83 g) was subjected to silica gel column chromatography (500 g, 200–300 mesh, Qingdao Marine Chemical Co. Ltd., Shandong, China) and eluted with the mixture of ethyl acetate and methanol. The active fraction (740 mg) was then subjected to Sephadex LH-20 column chromatography with methanol as the mobile phase. The obtained active ingredient was further purified on a Shimadzu 6AD HPLC apparatus (Shimadzu Co. Ltd., Tokyo, Japan) equipped with a column of Hypersil ODS-BP (20 × 250 mm, 10 μm, flow rate 8.0 mL/min) and eluted with the mixture of methanol and 0.1% acetic acid (6:4) to afford ambobactin (**1**) (19.8 mg).

### 3.4. Acid Hydrolysis and Advanced Marfey’s Analysis

Approximately 1.0 mg of ambobactin was hydrolyzed with 200 μL of 6 N HCl in a sealed high-pressure tube at 110 °C for 1 h [17]. The acid hydrolysate was evaporated to dryness and dissolved in 100 μL of distilled water. To each a half portion (50 μL) was added 80 μL of 1 N NaHCO_3_ and 100 μL 1% l-FDAA or d-FDAA (solution in acetone). The mixture was heated at 50 °C for 1 h. After cooling to room temperature, the reaction mixture was neutralized with 40 μL 1 N HCl, and evaporated to dryness. The residue was then dissolved in 0.2 mL acetonitrile and injected for HPLC-UV/MS/MS analysis. For 3-methyltryptophan analysis, ambobactin (0.2 mg) was treated with 6N HCl (0.1 mL) containing 0.05% phenol and 2% mercaptoethanol at 150 °C for 1 h [18].

### 3.5. HPLC-UV/MS Analysis

The LC-MS experiments were performed on the Finnigan LCQ Advantage Max ion-trap mass spectrometer (Thermo Fisher Co.) coupled with a surveyor HPLC. Separations were carried out on a Hypersil Gold column (150 × 2.1 mm) (Thermo Fisher Co.). Acetonitrile-0.1% trifluoroacetic acid was used as the mobile phase under a linear gradient elution mode (acetonitrile, 15%–70%, 40 min). The flow rate was 0.2 mL/min with UV detection at 340 nm. The sample solution from the outlet of the DAD detector was injected to the ESI interface directly. The ESI voltage was 5.4 KV with the sheath and auxiliary gas set at 60 and 5 arbitrary units, respectively, and the capillary was heated to 250 °C. A mass range of *m*/*z* 200–850 was covered, and data were collected in negative mode.

### 3.6. Tandem MS Analysis

To verify the amino acid sequence of ambobactin, tandem mass spectrometric analysis was carried out on the ion-trap mass spectrometer by a direct sample injection method. The quasi-molecular ion and other characteristic fragments were selected as parent ions to perform tandem MS analysis. The collision energy for MS/MS was adjusted to 35%, and the isolation width of precursor was set at 2.0 mass units.

### 3.7. Antibacterial Activity

The antibacterial activities of **1** against *B. cereus*, *B. subtilis*, *S. aureus*, *E. coli*, *P. aeruginosa*, *E. carotovora*, *P. syringae* pv. *actinidiae*, *A. solanacearum*, *X. oryzae* pv. *oryzae* were evaluated by the micro-broth dilution method in 96-well plates [17]. The inoculum was prepared by suspending several colonies from an overnight culture of tested bacteria from 0.5% sheep blood agar media in Müller-Hinton broth (Hangzhou Microbial Reagent Co. Ltd., Zhejiang, China) and adjusting to a 0.5 McFarland standard (approximately 1.5 × 10^8^ colony-forming units per mL). A further dilution of 1:200 was made by placing 0.25 mL of the adjusted suspension into 49.75 mL of Müller-Hinton broth. Compound **1** was firstly dissolved in DMSO at the concentration of 1 mg/mL, and it was diluted with sterile water to give the stock solution. Two-fold serial dilutions of the tested compound were prepared in Müller-Hinton broth. Then the dilutions and inoculated suspension of the bacteria were delivered to wells of a 96-well plate at the ratio of 1:1. The final concentration of inoculum in each well was 3.7 × 10^5^ colony-forming units per mL. After incubation for 24 h at 30 °C, minimum inhibitory concentrations (MICs) were examined. Experiments were repeated triplicate and standard ampicillin sodium was used as the positive control.

## 4. Conclusions

In summary, ambobactin (**1**), a novel cyclic depsipeptide, was isolated from the fermentation broth of *S. ambofaciens* F3. The only structural difference between telomycin and ambobactin is that the configuration of the 3-methyltryptophan moiety in these two compounds is different. Ambobactin exhibited broad antibacterial activity against both Gram-positive and Gram-negative bacteria. To our best knowledge, this is the first report that demonstrates *S. ambofaciens* is a producer of telomycin-like cyclic peptides.

## Supplementary Materials

Supplementary materials can be accessed at: http://www.mdpi.com/1420-3049/20/09/16278/s1.
