# Supplementary material for: Structure and Antibacterial Activity of Ambobactin, a New Telomycin-Like Cyclic Depsipeptide Antibiotic Produced by Streptomyces ambofaciens F3
Source: Molecules. 2015 Sep 9;20(9):16278–89. doi: 10.3390/molecules200916278 (PMC6331918; doi:10.3390/molecules200916278)
Supplement: Supplementary file 1 [file molecules-20-16278-s001.pdf]

Supporting Information

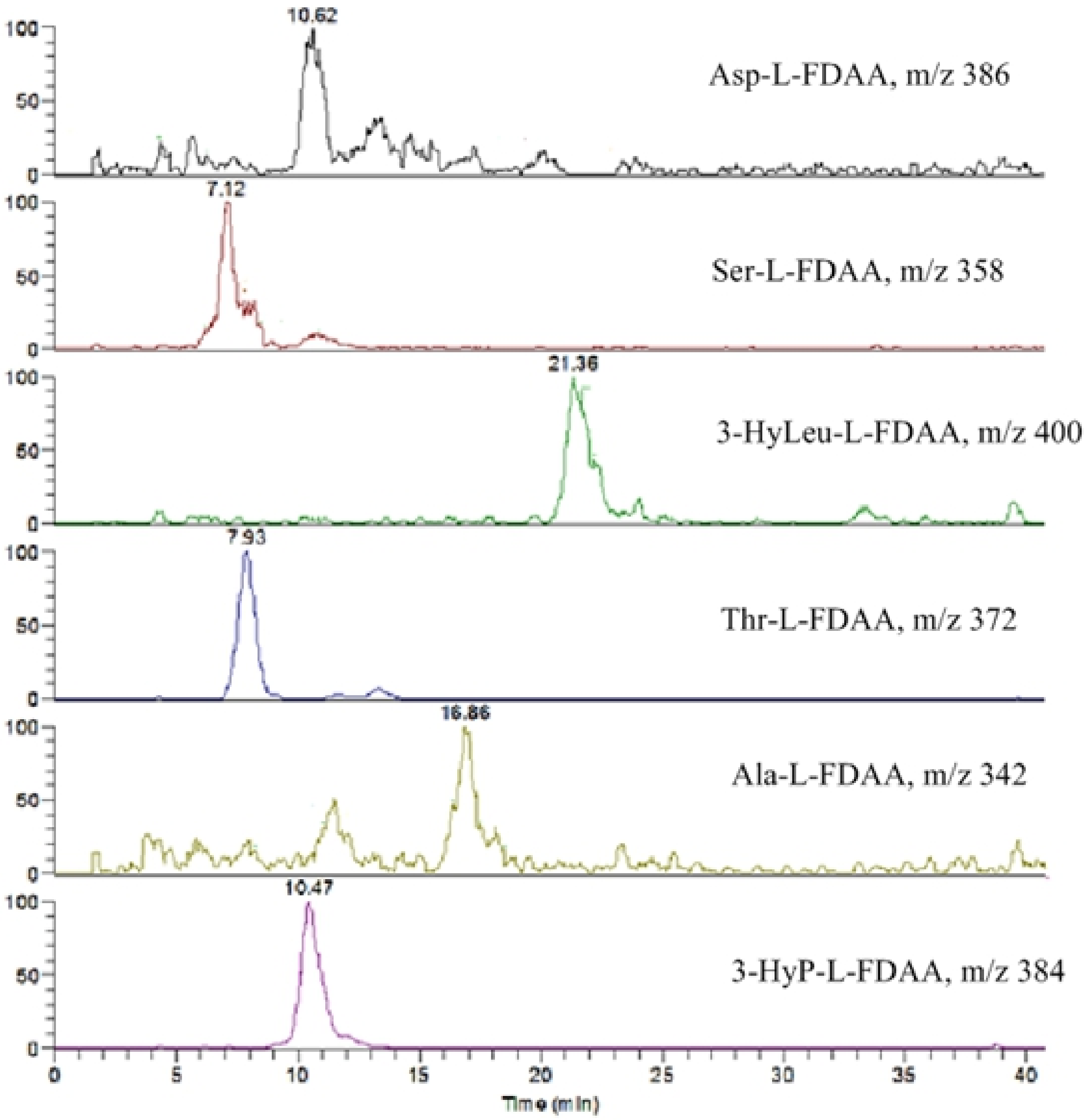

Figure S1. Selected ion chromatogram of L-FDAA derivatives.

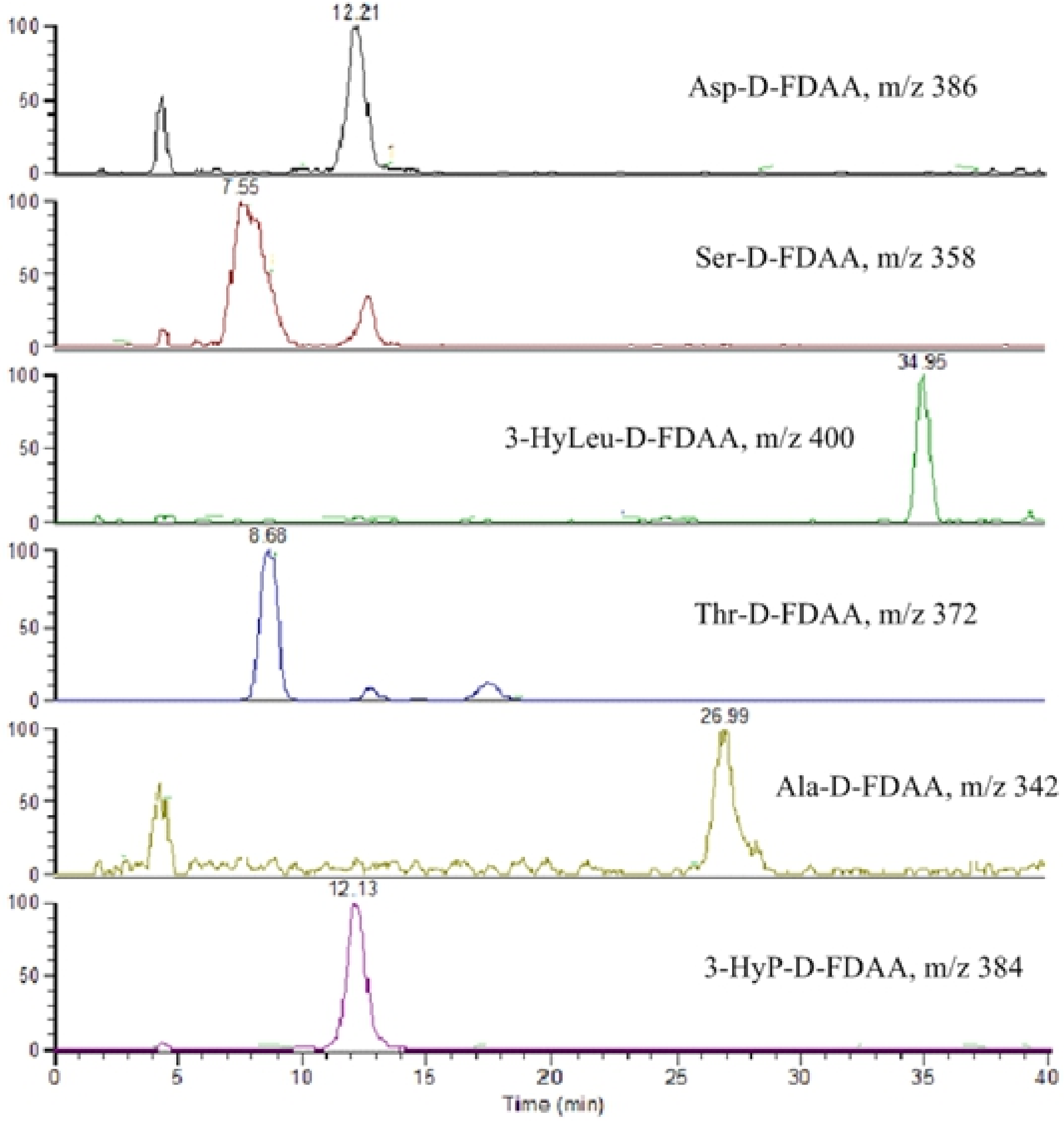

Figure S2. Selected ion chromatogram of D-FDAA derivatives.

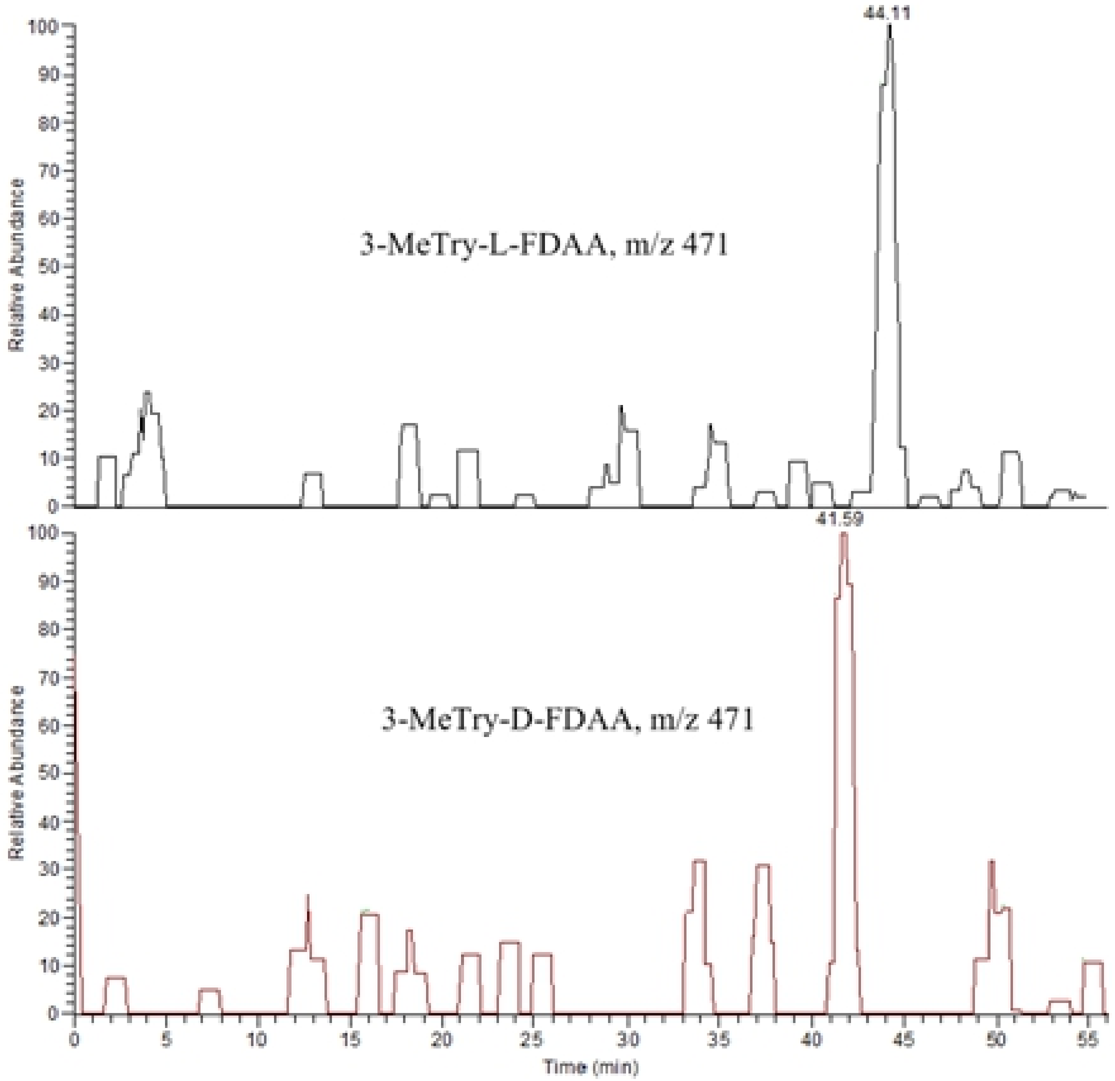

Figure S3. Selected ion chromatogram of L- and D-FDAA derivatives of 3-MeTry.

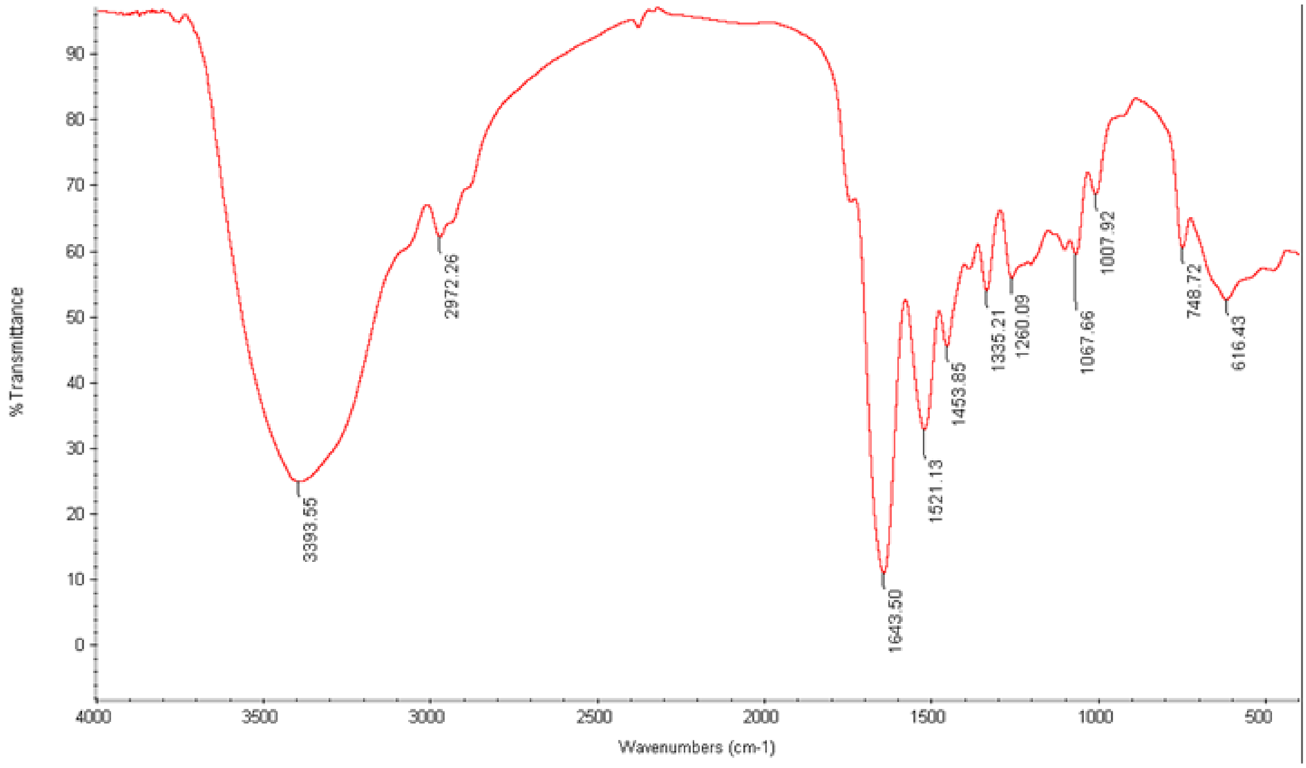

Figure S4. IR spectrum of **1**.

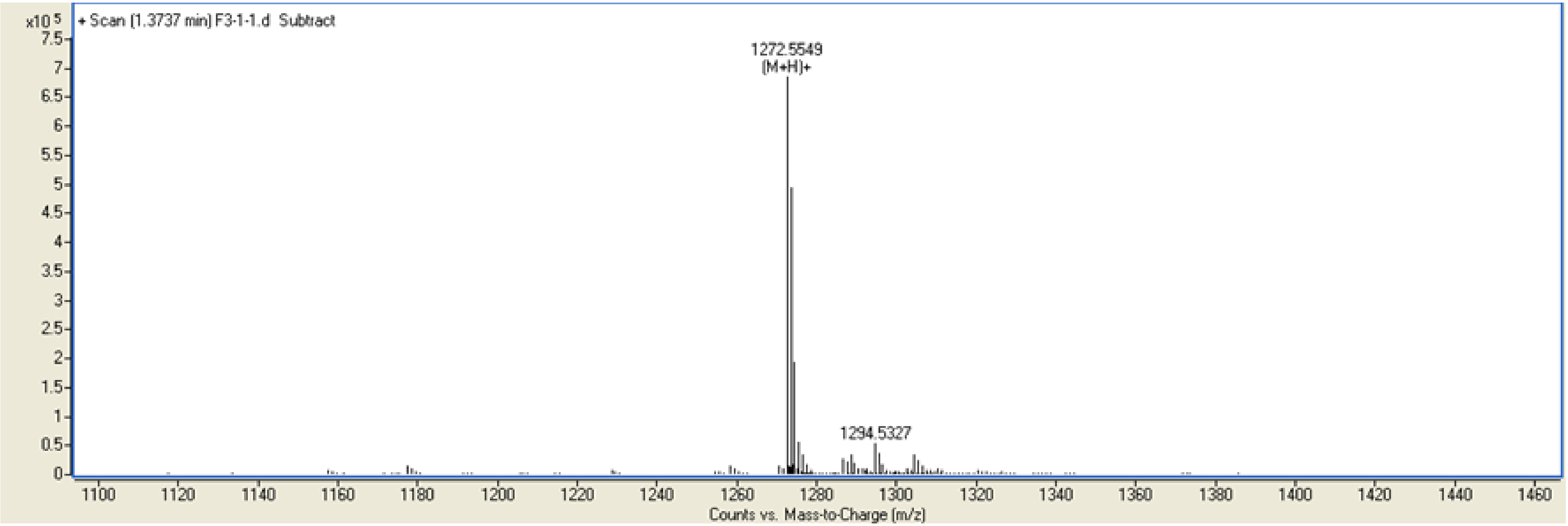

Figure S5. HRMS spectrum of **1**.

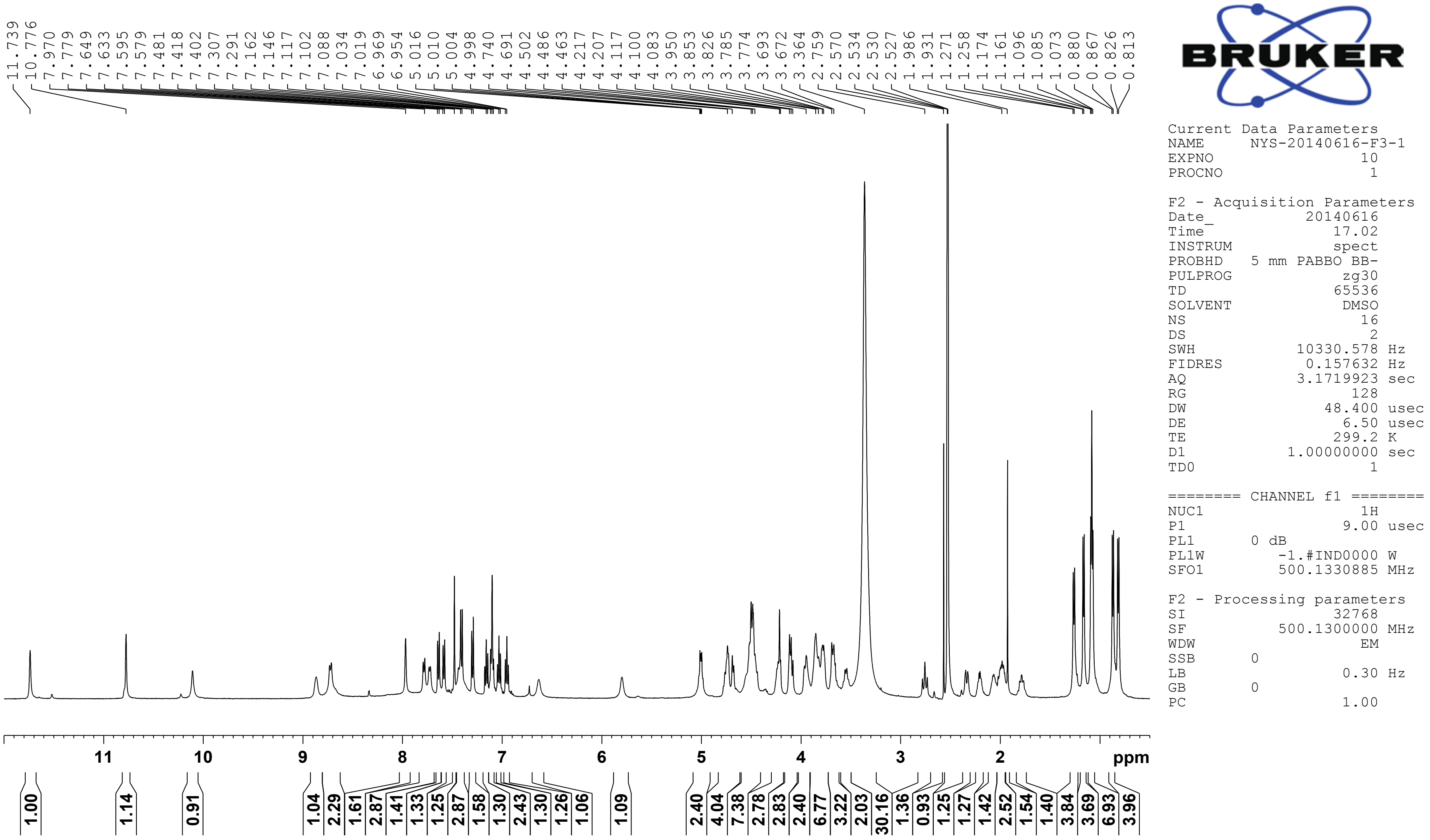

Figure S6. ^{1}H-NMR Spectrum of **1** in DMSO.

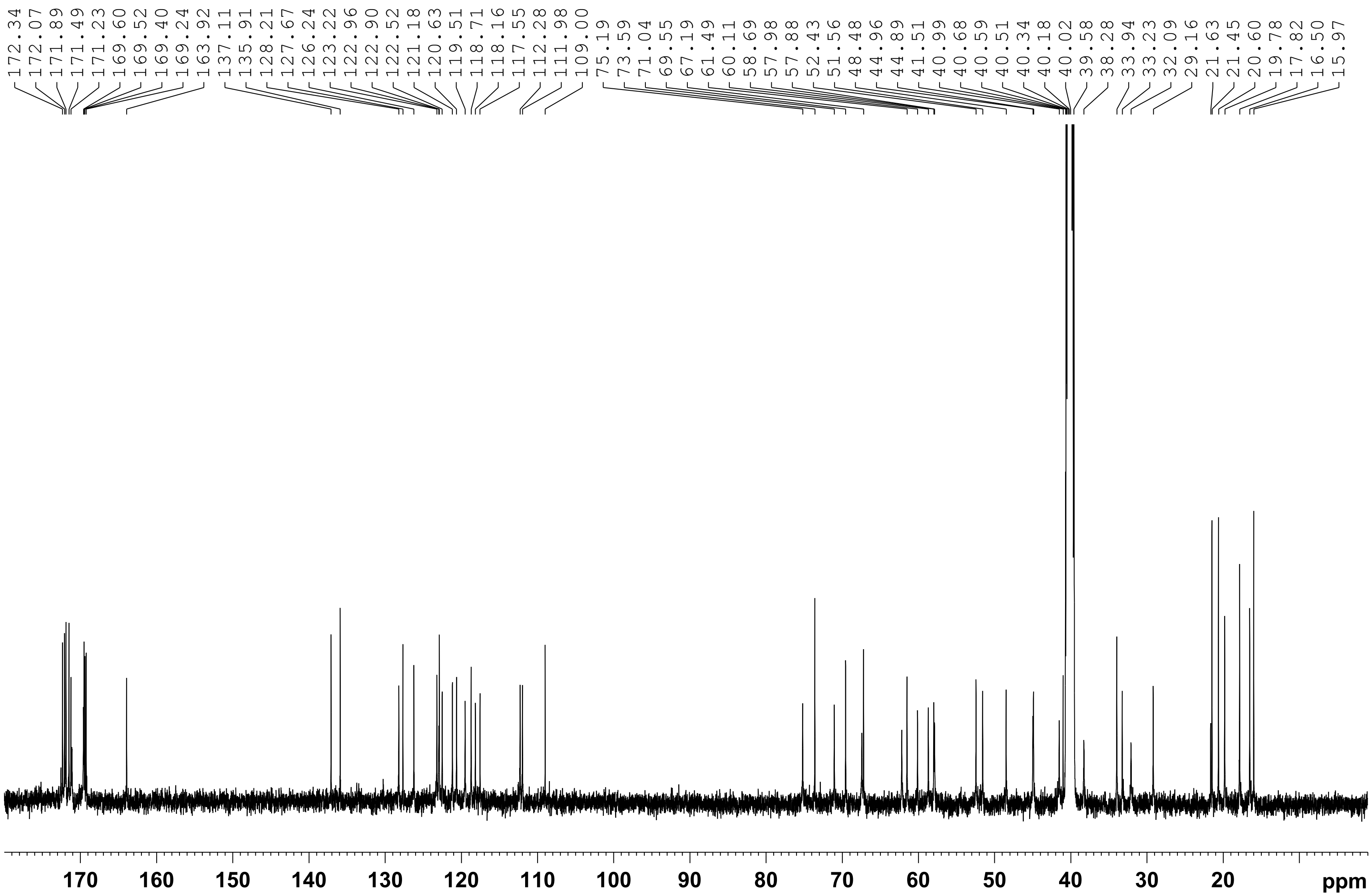

Figure S7. ^{13}C-NMR Spectrum of **1** in DMSO.

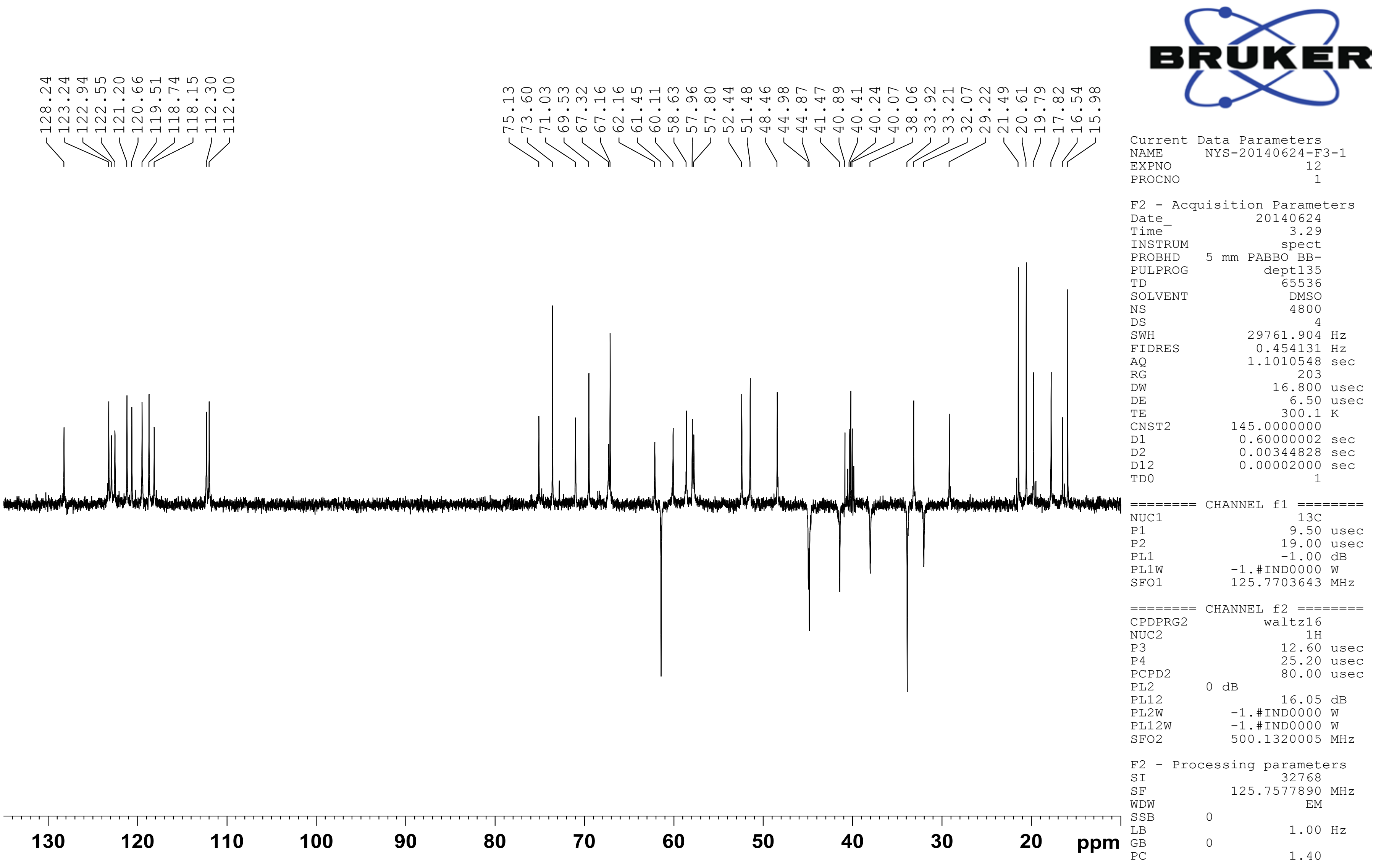

Figure S8. DEPT-135 Spectrum of **1** in DMSO.

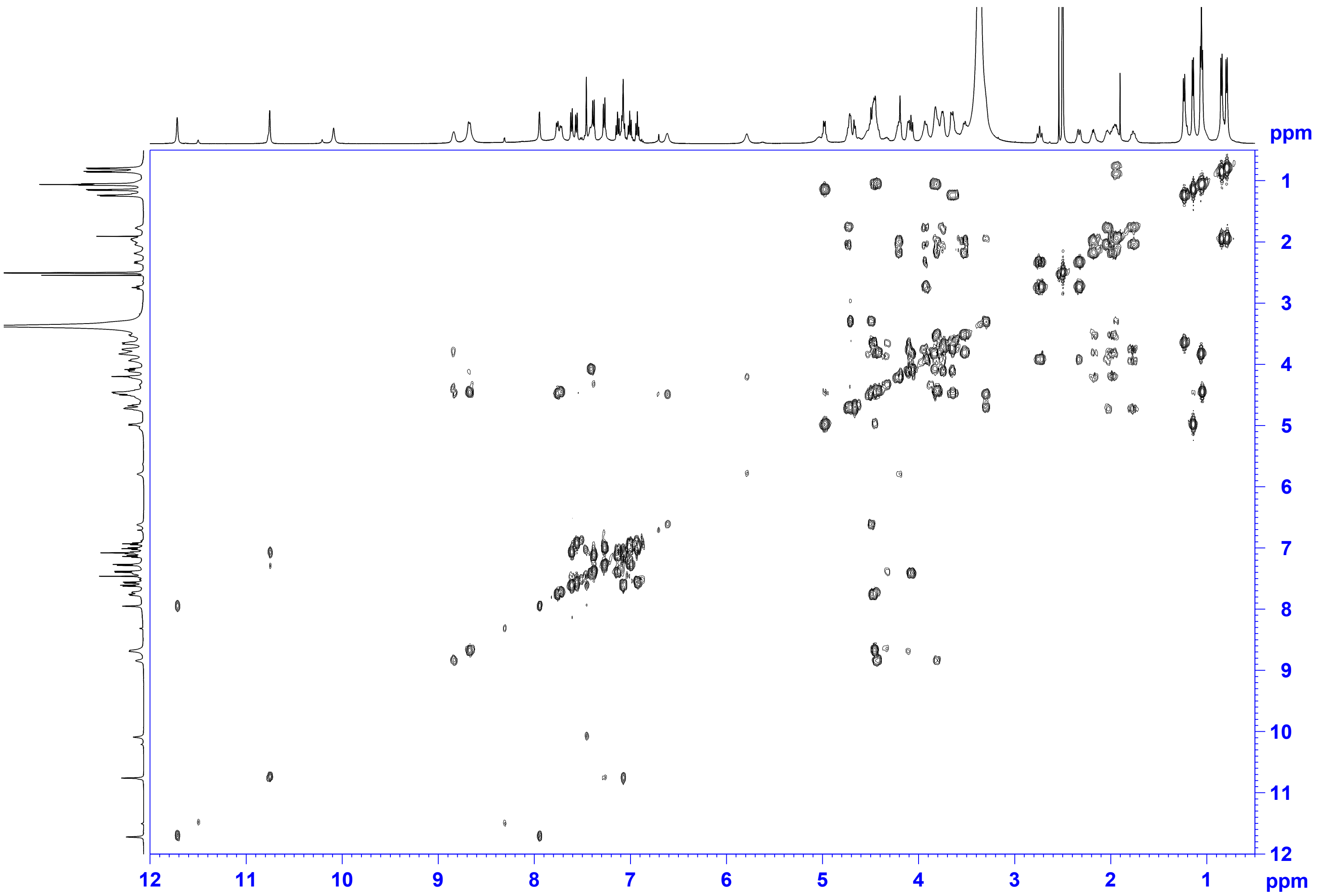

Figure S9. ^{1}H, ^{1}H-COSY Spectrum of **1** in DMSO.

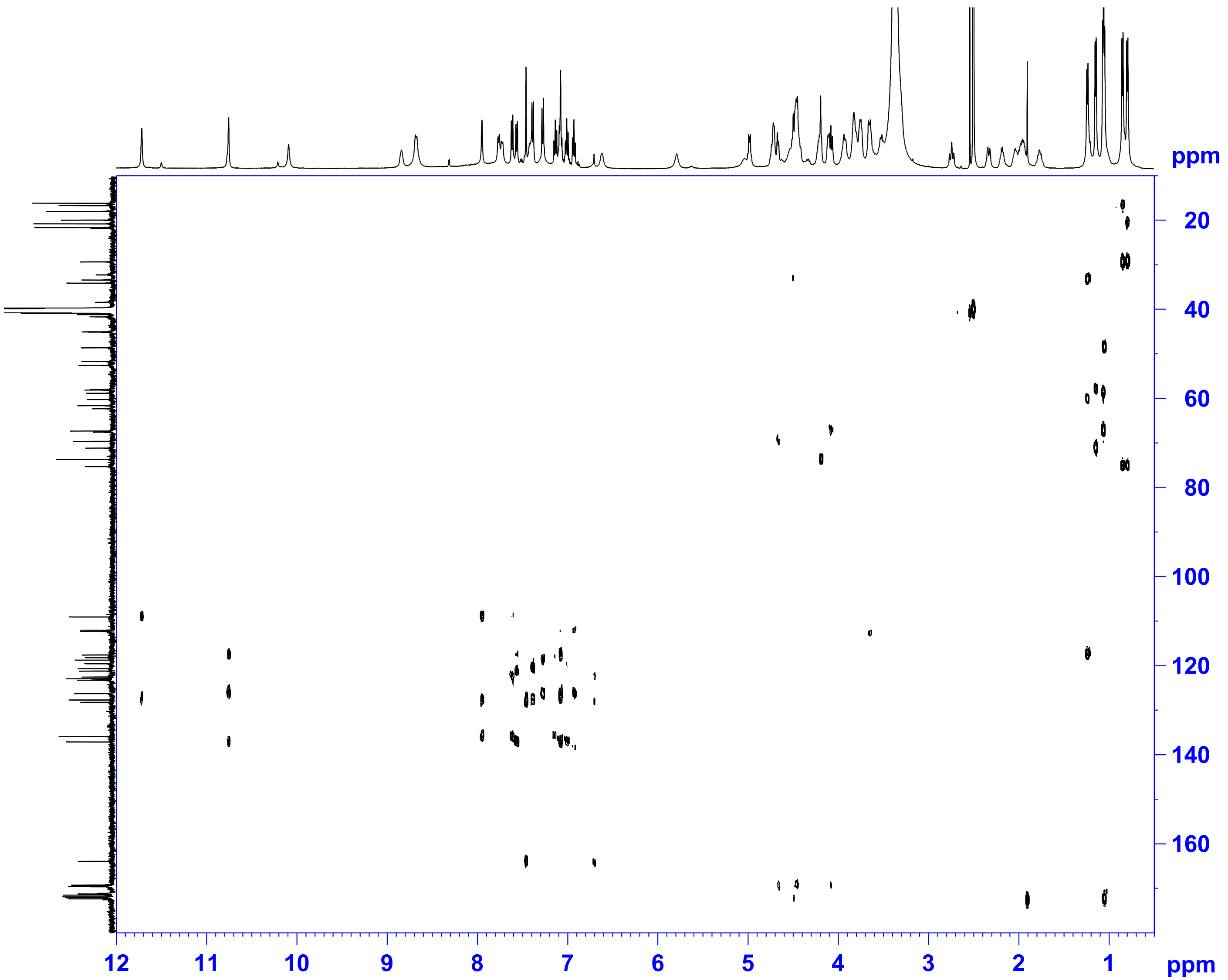

Figure S10. HMBC Spectrum of **1** in DMSO.

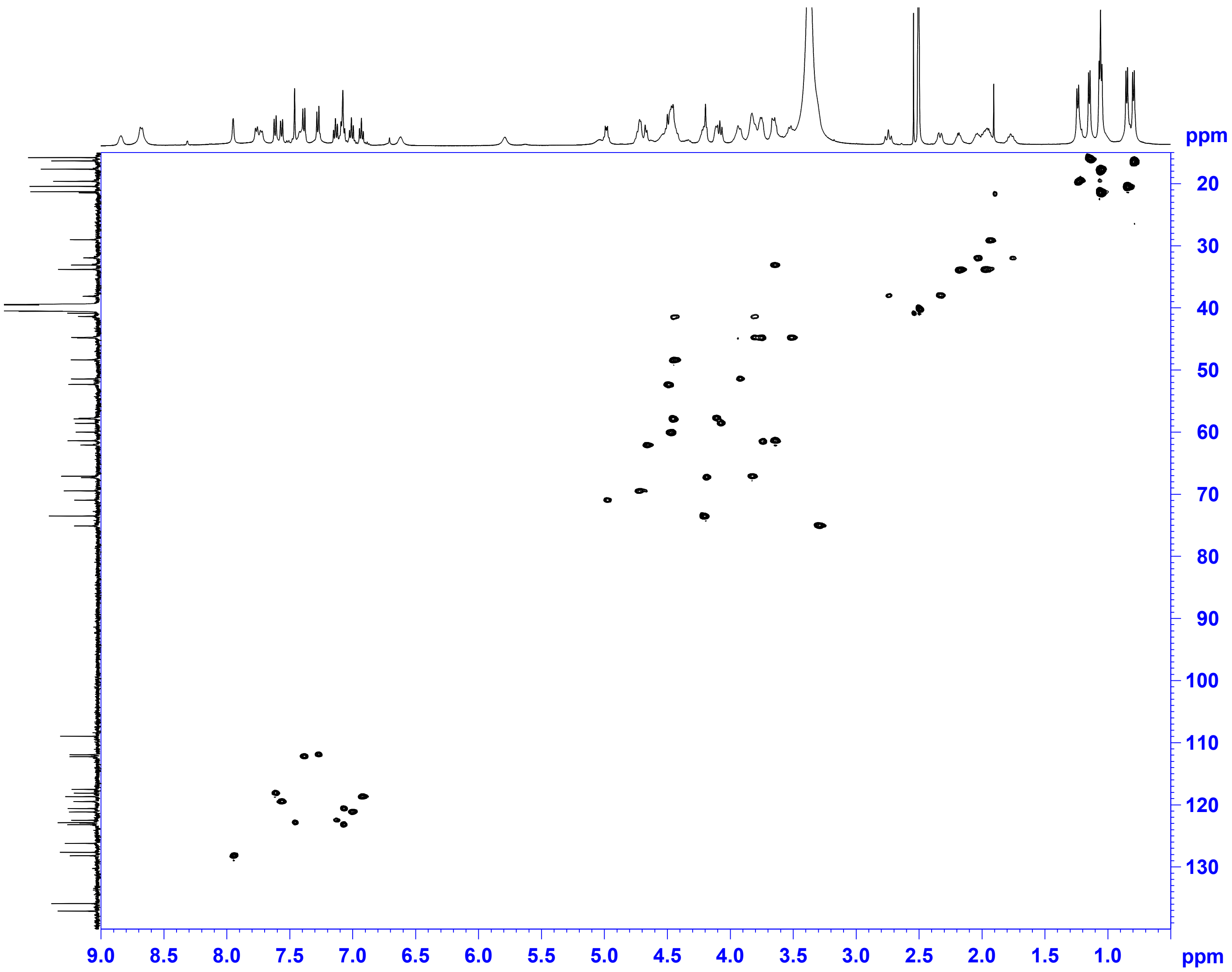

Figure S11. HSQC Spectrum of **1** in DMSO.

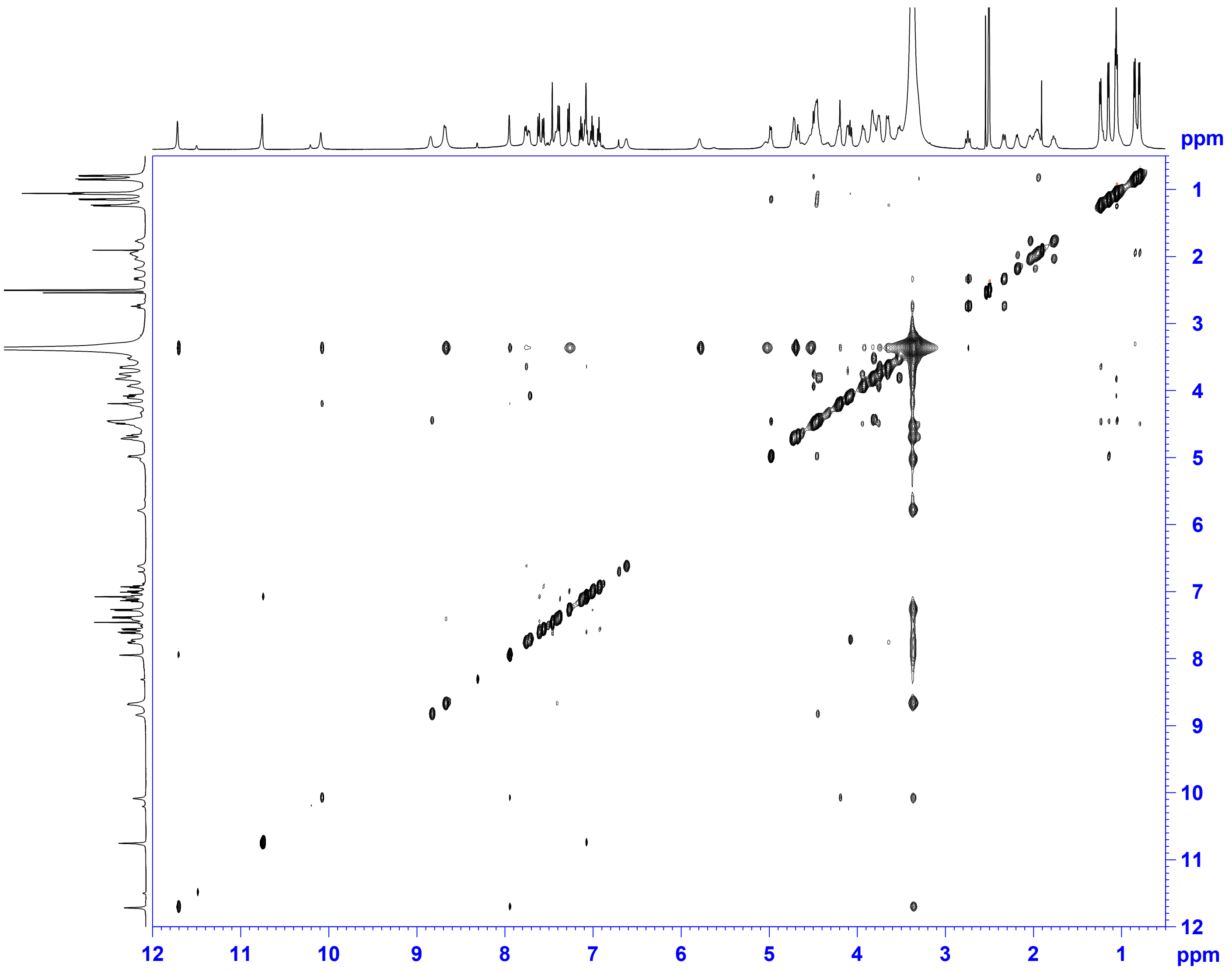

Figure S12. NOE Spectrum of **1** in DMSO.
